# Supplementary material for: Characterization of Brucella abortus Mutant A19mut2, a Potential DIVA Vaccine Candidate with a Modification on Lipopolysaccharide
Source: Vaccines (Basel). 2023 Jul 21;11(7):1273. doi: 10.3390/vaccines11071273 (PMC10385478; doi:10.3390/vaccines11071273)
Supplement: Supplementary file 1 [file vaccines-11-01273-s001.zip › vaccines-2466966-supplementary.pdf]

Supplementary:

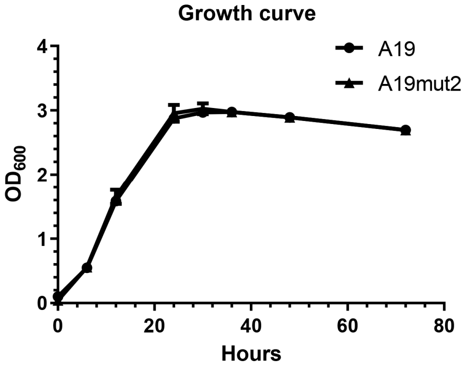

Figure S1. The growth curves of the A19 and A19mut2 strains.

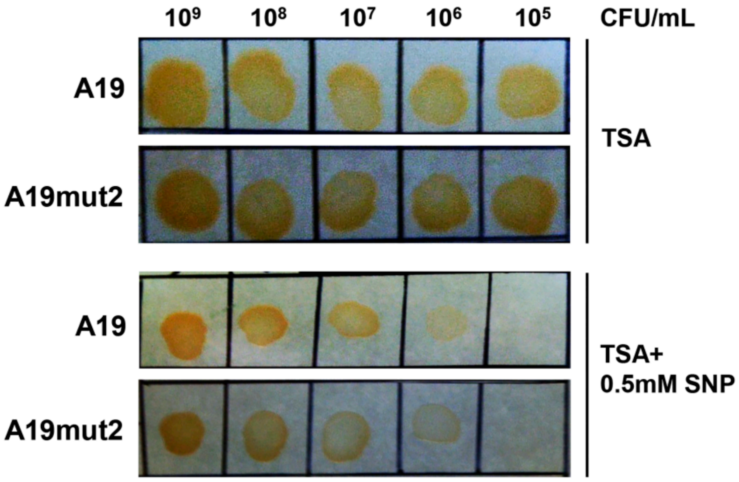

Figure S2. The resistance of the A19 and A19mut2 to sodium nitroprusside.
